# The neXtProt knowledgebase on human proteins: current status

**DOI:** 10.1093/nar/gku1178

**Published:** 2014-12-12

**Authors:** Pascale Gaudet, Pierre-André Michel, Monique Zahn-Zabal, Isabelle Cusin, Paula D. Duek, Olivier Evalet, Alain Gateau, Anne Gleizes, Mario Pereira, Daniel Teixeira, Ying Zhang, Lydie Lane, Amos Bairoch

**Affiliations:** 1 CALIPHO group, SIB Swiss Institute of Bioinformatics, Geneva, Switzerland, 1211; 2 Department of Human Protein Sciences, Faculty of Medicine, University of Geneva, Geneva, Switzerland, 1211

## Abstract

neXtProt (http://www.nextprot.org) is a human protein-centric knowledgebase developed at the SIB Swiss Institute of Bioinformatics. Focused solely on human proteins, neXtProt aims to provide a state of the art resource for the representation of human biology by capturing a wide range of data, precise annotations, fully traceable data provenance and a web interface which enables researchers to find and view information in a comprehensive manner. Since the introductory neXtProt publication, significant advances have been made on three main aspects: the representation of proteomics data, an extended representation of human variants and the development of an advanced search capability built around semantic technologies. These changes are presented in the current neXtProt update.

## INTRODUCTION

neXtProt (http://www.nextprot.org) is a web-based knowledge platform focusing on human proteins. Similarly to Model Organism Databases (MODs) which serve to collate data and provide an impetus for research on model species, the goal of neXtProt is to serve as a one-stop shop for research on human proteins by providing a representation of the current state of knowledge in a manner that is at once both comprehensive and of high quality. Since the first publication on neXtProt (1), we have continued to expand the database. We have developed close collaborations with two major user groups: proteomics researchers, who use mass spectrometry techniques to identify the different protein forms present in biological samples and biomedical researchers working on elucidating how genetic variations in protein-coding sequences can lead to disease. Our recent work has been mostly focused on integrating data from these two areas of human biology, with extensive quality control procedures. Major efforts have been undertaken on the search and retrieval capacities of neXtProt in order to take into account the richness of annotations and evidences so as to support the retrieval of proteins based on highly precise criteria as well as to allow programmatic data access. The next sections of this paper describe these improvements in detail.

## NEW neXtProt CONTENT

neXtProt continuously adds new content to the database. Table 1 displays the information contained in neXtProt as of October 2014. The major data sources include Bgee (2), HPA (3), Peptide Atlas (4), SRMAtlas (5), UniProtKB (6), GOA (7), dnSNP (8), Ensembl (9), COSMIC (10), DKFGFP-­‐cDNA localization (11,12), Weizmann Institute of Science's Kahn Dynamic Proteomics Database (13), andIntAct (14).

**Table 1.** neXtProt contents in the October 2014 release

Data type	Number of annotations^a^	Source
Anatomical (cell types/tissues/organs) expression	1 823 655 Gold/1 263 848 Silver	Bgee (microarray and ESTs) (2) and HPA (immunohistochemistry) (3)
Mass spectrometry-identified peptides	1 090 163, all Gold	PeptideAtlas (4), SRMAtlas^b^ (5), and direct integration of results of research articles
Post-translational modifications	94 019 Gold/8575 Silver	UniProtKB (6) and direct integration of results of research articles
Gene Ontology annotations	136 852 Gold/54 712 Silver	GOA (7)
Variants	70 262 Gold/1 081 000 Silver	UniProtKB, dbSNP (8) (via Ensembl (9)), COSMIC (10)
Subcellular localizations	26 148 Gold/8582 Silver	UniProtKB, HPA, GOA, DKF GFP-cDNA localization (11,12); and Weizmann Institute of Science's Kahn Dynamic Proteomics Database (13)
Interactions	9467 Gold/71 911 Silver	UniProtKB and Intact (14)

^a^Gold and Silver quality assignment varies by data source and has been set in accordance with data providers whenever possible (1); see also (21) for quality assignment regarding HPA.

^b^New in the October 2014 release.

In addition to the data presented in Table 1, neXtProt provides (i) mappings of proteins to their Ensembl genomic transcripts on the human genome; (ii) associations with over 800 000 identifiers, including cDNA clone names encoding for the proteins, Affymetrix and Illumina DNA probe sets; (iii) cross-references to CCDS (15), HPRD (16); and (iv) abstracts of all articles from PubMed that are cited in human UniProtKB/Swiss-Prot entries as well as some cited by other resources such as Entrez Gene (GeneRIFs) (17), MINT (18), and PDB (19) and which have been computationally mapped to the relevant protein entry by the UniProt consortium, totaling over 400 000 references. We have also recently integrated a 3D structure visualization applet—BioViz—developed by BIONEXT (http://www.bionext.com). The current version of the applet allows users to zoom, select regions or position-specific annotations (such as post-translational modifications (PTMs)) and view them in the context of the 3D structure, in addition to highlighting them in the graphic, table and sequence views of the Structures page for an entry.

## FOCUS ON PROTEOMICS

HUPO, the Human Proteome Organization (http://www.hupo.org), is an international group that connects all laboratories using proteomics as an approach to characterize human proteins in healthy and disease samples. HUPO's Human Proteome Project (HPP; http://www.thehpp.org (20)) aims to make a comprehensive inventory of all proteins with respect to their existence, the different isoforms expressed, post-translational modifications as well as their abundance, distribution and subcellular localization. neXtProt has been selected as the knowledge resource for the HPP project (21). As such, neXtProt's role within the HPP project is to integrate the results of the mass-spectrometry (MS) identification studies that are flagged as being part of HPP; provide metrics concerning the progress of the project (which proteins still need to be identified by proteomics); and represent the extent of our knowledge of human proteins’ properties and functions in the best possible manner.

**Figure 1.**
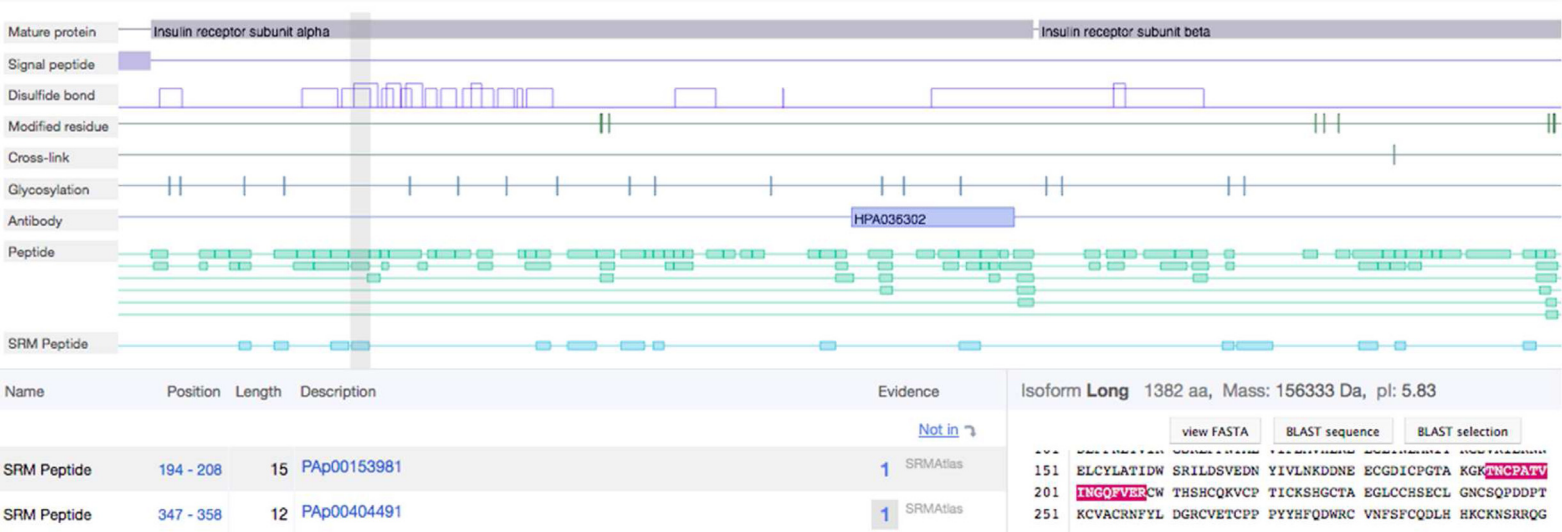
The neXtProt proteomics view displays a new track for ‘SRM Peptides’ that have been chemically synthesized and validated by SRMAtlas. As for the other views displaying sequences, the graphical view, the table and the sequence are linked together, so that upon selection of a peptide in the graphical view, it is highlighted in the table and in the sequence. As shown by the peptide selected above, some SRM peptides correspond to natural peptides identified in biological samples; in this case they are shown twice, with their respective evidences.

PeptideAtlas (5), developed at the Seattle Proteome Center, is a close collaborator on the HPP project (4). PeptideAtlas collects raw results from proteomics experiments and reinterprets them using a uniform computational pipeline, the Trans-Proteomic Pipeline (22), with a stringent false-discovery rate cut off of 1%. PeptideAtlas provides peptide identification in biological samples, i.e. protein existence validation. PeptideAtlas has proteomics data from multiple tissues and fluids: plasma, urine, brain, kidney, heart, liver, lung, digestive system, pancreas, spleen, eye, breast, adrenal gland, urinary bladder and female and male reproductive systems. On its proteomics page, neXtProt presents peptides identified in experiments integrated by PeptideAtlas. Moreover, neXtProt displays the tissues in which a peptide was identified in the Evidences column of the table view. Another project of the Seattle Proteome Center is the SRMAtlas, an atlas of peptides detected by Selected Reaction Monitoring (23). This technique is currently the most precise method for quantifying peptides by mass spectrometry. SRMAtlas provides tools (i.e. synthetic peptides spectra) to allow protein identification and quantitation in biological samples. As of October 2014, neXtProt displays the peptides validated by SRMAtlas (Figure 1).

neXtProt also integrates data directly from high-throughput studies. We have integrated 21 papers with post-translational modifications, covering several different types of modifications: phosphorylation, N- and O-glycosylation, sumoylation, ubiquitylation, acetylation and methylation. Again, only high quality data is loaded, based on stringent criteria that vary from paper to paper, but that usually require a protein false discovery rate (FDR) of 1% of less.

## neXtProt EXTENDS THE COVERAGE OF IDENTIFIED PROTEINS IN THE HUMAN PROTEOME

As described previously (21), neXtProt uses data from UniProtKB and from proteomics studies to assign levels of evidence for protein existence applying the same criteria as UniProtKB: (i) evidence at protein level (e.g. identification by mass spectrometry, or detected by antibodies, or sequenced by Edman degradation, or that its tridimensional structure has been resolved, (ii) evidence at transcript level (e.g. ESTs or full length mRNA), (iii) inferred by homology (strong sequence similarity to known proteins in related species), (iv) predicted (gene models) and (v) uncertain (e.g. dubious sequences that are likely the products of erroneous translations of pseudogenes). The October 2014 release of neXtProt contains 16 491 entries validated at the protein level out of 20 055 entries, or 82%, compared with 15 603 in the October 2013 release, a 4% increase. The UniProtKB release 2014_08 contains 13 988 human entries validated at the protein level. Thus, the integration of additional proteomics data has meant that neXtProt has integrated experimental evidence for the existence of 2503 additional entries.

## FOCUS ON VARIANTS

Across the whole spectrum of human population, there are millions of variations in protein sequences (24), most of which having no consequence on health. However a great challenge that derives from easier access to exome and whole genome sequencing is trying to identify those mutations that may cause a pathologic effect or increase the risk to certain diseases. With our expertise on human protein function, we have embarked on a project of annotation of protein variants implicated in hereditary cancers. To do so, we are developing an annotation platform to annotate protein function and mutant phenotypes, which is still at the prototypical stage and will be presented in a future publication. In order to annotate protein variants as exhaustively as possible, we have integrated mutations from the COSMIC database (10), and are in the process of integrating those from ClinVar (25). The variants we are integrating are those that affect protein sequence, and are of type: substitution, insertion and deletion.

### Disease and cell line mappings

neXtProt strives to support interoperability with other resources by using standard vocabularies and ontologies whenever possible. When this is not possible, we construct vocabularies and mappings that we make publicly available on our FTP site. COSMIC uses its own internal classification system to describe diseases and cell lines. This led us to develop two resources, the Cosmosaurus and the Cellosaurus, to address this issue.

### The Cosmosaurus: a mapping between COSMIC and the NCI Thesaurus

We have created a mapping between COSMIC and the NCI Thesaurus (26). This mapping is named ‘Cosmosaurus’. In COSMIC, each sample is described using four fields: ‘Primary site’, ‘Site subtype’, ‘Primary histology’ and ‘Histology subtype’. The Cosmosaurus treats each distinct combination of these four fields as a synonym (SY) for a NCI entry, defined by its NCI Thesaurus term (ID) and accession (AC). The mapping was developed in-house, with the invaluable help of COSMIC biocurators. An example of a Cosmosaurus mapping is shown below. In this case, four different combinations of COSMIC sample descriptions are mapped onto a single NCI term. The version of July 2014 contains 1706 COSMIC terms mapped to 736 NCI Thesaurus terms.

**Table:** 

ID	Colorectal Tubular Adenoma
AC	C27456
SY	large intestine, caecum, adenoma, tubular
SY	large intestine, left, adenoma, tubular
SY	large intestine, NS, adenoma, tubular
SY	large intestine, right, adenoma, tubular

### The Cellosaurus: an extensive glossary of cell lines

Many of the data annotated in COSMIC come from cell lines. Unfortunately, there was no comprehensive standardized resource describing cell lines. To address this, we have developed a new controlled vocabulary, the Cellosaurus, which, as far as we know, is the most comprehensive resource on cell lines (Bairoch, *in preparation*). The Cellosaurus is constantly growing; the version of September 2014 contains over 32 000 cell lines from 240 species (74% from human, 14% from mouse), 21 000 synonyms and 23 000 publication references to over 6500 publications. COSMIC samples originating from a cell line are mapped to a Cellosaurus unique identifier.

## FOCUS ON SEARCH AND DATA RETRIEVAL

**Figure 2.**
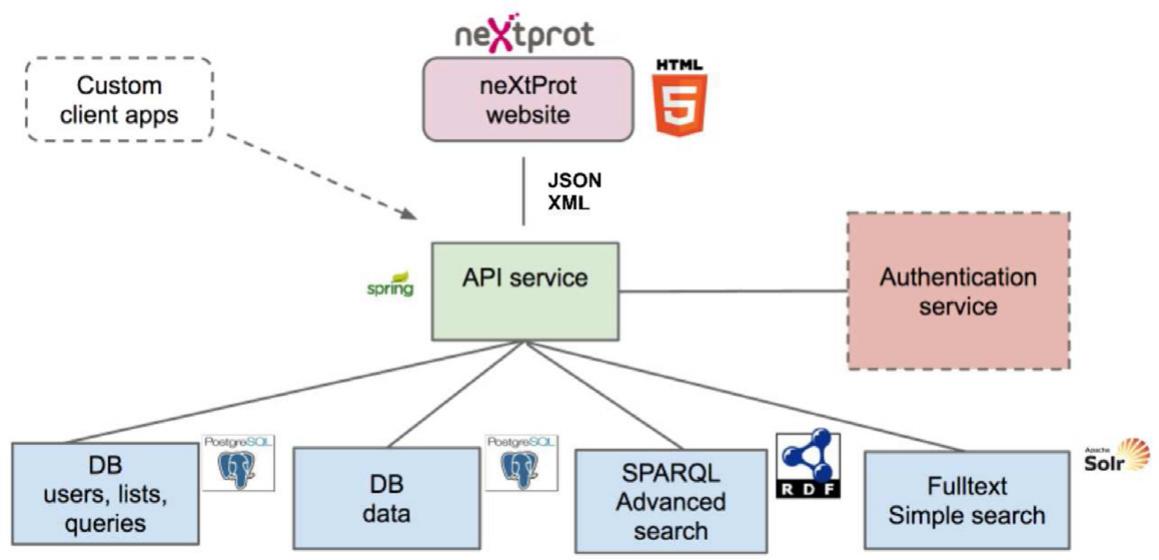
The new neXtProt infrastructure. neXtProt implements the following software and packages: *Spring:* open source web application over Java (spring.io); *Spring JDBC templates:* thin API between database and data objects (http://docs.spring.io/spring/docs/3.0.x/spring-framework-reference/html/jdbc.html); *PostgreSQL:* database where sequences, annotations, evidences and terms, as well as user resources (profiles, saved lists and queries) are stored (http://www.postgresql.org); *Lucene/solr:* full-text search engine for simple search queries (http://lucene.apache.org and http://lucene.apache.org/solr); *Jena:* graph search API for complex search queries (http://jena.apache.org); and *Virtuoso:* to store RDF data and perform complex SPARQL search queries (http://virtuoso.openlinksw.com).

We have completely restructured the neXtProt data model and infrastructure. The objective of these changes was to allow neXtProt users to precisely extract information that they are interested in; to manage list of proteins (originating from the results of searches, or created by users); and build analysis tools on top of neXtProt. Figure 2 gives an overview of the new neXtProt architecture and the technologies used.

With the new architecture, all the data in neXtProt is now accessible via a REST API. The REST API decouples the database from all our services; in particular, the search and the export services. Importantly for our users, the REST API provides an easy access to all data such that third parties can build applications on top of neXtProt.

**Figure 3.**
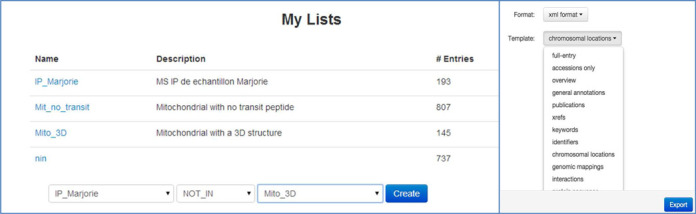
The neXtProt list manager can be used to save lists, make operations such as combining them, and export the entries contained in a list. The full entries can be exported, or only certain data, such as accession numbers, the overview, the general annotations, etc.

Results from neXtProt searches are linked to a protein list management tool (Figure 3). Users can create new lists, either from search results or by entering their own list; combine lists using the Boolean operators ‘AND’, ‘OR’ and ‘NOT IN’; find common items between two lists. Lists can be saved and used for further operations, for example export. Entries can be exported in their entirety, or the user can customize which content they wish to export, for instance the sequence or a subset of annotation types like PTMs or expression profiles.

**Figure 4.**
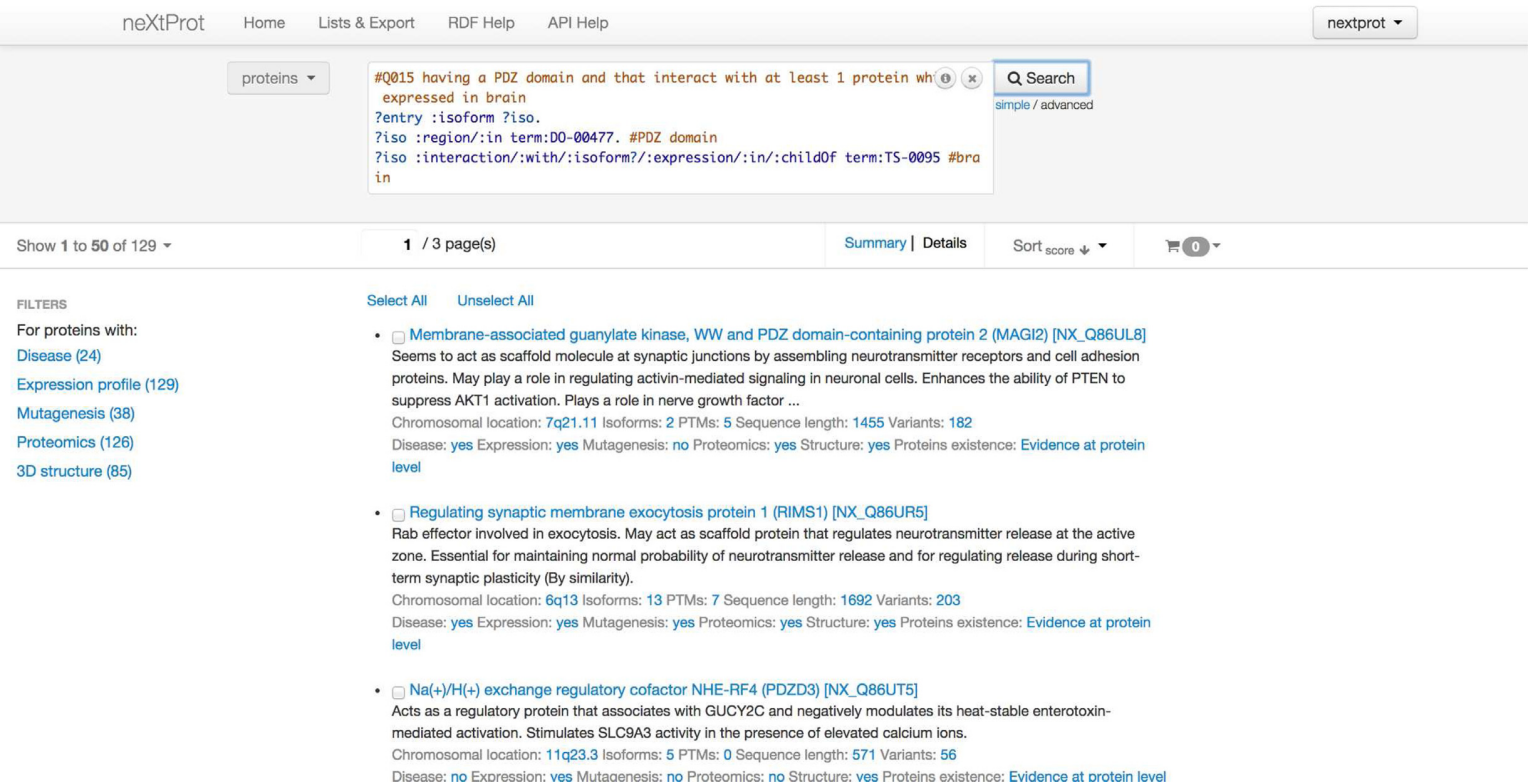
Example of a neXtProt advanced search. This query retrieves entries corresponding to proteins having a PDZ domain and that interact with at least one protein expressed in the brain. The query returns 129 entries. Results can be sorted according to gene name, protein name, protein family name, chromosome, accession number or protein length. The results can also be saved as list, and that list exported in different formats such as text, XML or JSON.

A major advance in the neXtProt functionality is the availability of a new advanced search system, designed to support the retrieval of proteins based on highly precise criteria taking into account the richness of the annotations and evidences. To implement this new functionality, we have converted the relational database into a graph representation using a subject-predicate-object model, RDF-based (Resource Description Framework). The graph representation is extremely powerful to navigate through the richness of the neXtProt data and to search it using the SPARQL query language. The advanced search is accessible at http://search.nextprot.org. An example query is shown in Figure 4.

The syntax of the SPARQL query language is admittedly complex. We have made efforts to remedy to this by choosing predicate names that are as intuitive and distinctive as possible. We also plan to add pre-calculated ‘shortcut’ predicates. For instance, predicates expressing positional relationships between features (such as: *next_to, overlaps_with, upstream, downstream*, etc.) would improve readability, expressivity and performance of queries.

**Figure 5.**
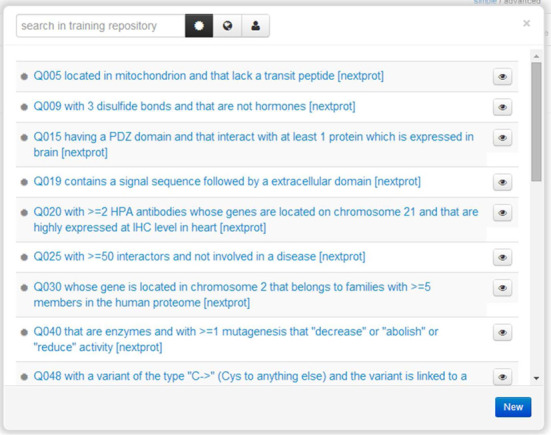
Examples of SPARQL queries available in the advanced search page. The examples cover a wide range of different queries and many incorporate counts of objects (for example, retrieve proteins having three disulfide bonds).

Moreover, to assist users construct queries, we provide a help page describing the data model where the domain and range of each predicate is given as well and a list of key resources (quality qualifiers, data sources, protein existence levels, etc.). We also provide a wealth of examples of queries that can be used directly or modified by users. Users can also save their queries; and keep those either private or make them available publicly. Examples of queries are shown in Figure 5.

The advanced search will also be available via a SPARQL endpoint (http://api.nextprot.org/sparql). Using a SPARQL-based technology also allows performing federated queries with external resources that also offer a SPARQL endpoint. We provide examples of federated queries with DrugBank (http://www.drugbank.ca (27)) and with UniProtKB.

## FOCUS ON QUALITY

neXtProt aims to be both comprehensive and of high quality. Hence, although we aim to integrate as much relevant data as possible, the datasets are carefully selected and the quality of the data determined upon loading in the database; data deemed of low quality is excluded by data filtering. For each dataset and controlled vocabulary integrated in neXtProt, spot checks covering all types of the data are performed in order to ensure that the data is properly parsed, displayed in our web site and present in the export files. Problems identified in a dataset are immediately communicated to the data source contributing to a virtuous circle and resulting in improved data quality. Many checks are also performed at each neXtProt release to ensure data integrity and retrievability, tool functionality as well as the proper implementation of new features.

## DATA AVAILABILITY

Like any other neXtProt annotation, the variant data is available in our export files in XML and PEFF formats (described in (1)) on our FTP site (ftp://ftp.nextprot.org/). They can also be accessed from our API at http://api.nextprot.org. This content is available under the Creative Commons Attribution-NoDerivs License.

## CONCLUSIONS

neXtProt is being built as a participative platform and we look forward to receiving users’ input for the future development of neXtProt. Next developments include the continued expansion of the types of data captured in neXtProt. We also wish to support users who will take advantage of our API to incorporate some of neXtProt data into new bioinformatics applications. This will allow these applications to benefit from our efforts in providing high-quality curated knowledge on human proteins.
